# Book Review

**Published:** 1895-03

**Authors:** 


*The Rectum and Anus.* By CHARLES B. BALL. Second Edition. Pp. xiii., 439. London: CASSELL AND COMPANY, LIMITED. 1894. —We took occasion to praise the first edition of this clinical manual when it appeared six years ago; and we now welcome the second edition, which has been largely re-written so as to embrace most of the modern developments in this department of surgery, in which there has been perhaps more advance during recent years than in any other. Dependent on the discovery by Symington of little muco-cutaneous processes called anal valves, a new explanation is given of the formation of irritable ulcer of the anus. A good description is given of the various operations for excision of the rectum, including Kraske's operation, and the one usually adopted by the author by the method of trans-sacral incision. The book is well written throughout. There is not a superfluous sentence. Much care has been taken to include the recent work of German surgeons, while due appreciation is shown to well-known British authorities such as Allingham, Cripps, Bryant, and Treves. This edition well maintains the high reputation of Cassell's clinical manuals.

*The Common Forms of Dyspepsia in Women.* By ROBERT SAUNDBY, M.D. Pp. 33. Birmingham: CORNISH BROTHERS. 1894.— This volume, containing the Ingleby Lectures of 1894, directs attention to the proposition that the greater part of the stomach troubles which occur in women originate in a neurosis which depends upon general exhaustion from anæmia or overwork. The subject is of much interest, and has an important practical value. The lectures are well worthy of careful study.

*Analyses of Twelve Thousand Prescriptions.* Compiled by W. MARTINDALE. London: H. K. LEWIS. 1894.—The compiler of these statistics, with a view to the provision of facts for the revision of the *British Pharmacopœia*, has endeavoured to ascertain what changes have occurred during the last decade either from advances in medical treatment or improvements in the art of pharmacy. Many of the official preparations have not been used, whilst a long list of unofficial preparations has been much in demand. The highest scores have been spiritus chloroformi and tinctura nucis vomicæ.

*The Harrogate Mineral Waters.* By ARTHUR ROBERTS, M.D. Pp. 48. Harrogate: R. ACKRILL. 1894.—Recent analyses of the waters have been made, and the Corporation are spending large sums in making new baths, a winter garden, and a covered promenade. Harrogate is now able to compete with any foreign spa where sulphur and iron waters are useful for the cure of disease: eighty springs of water containing sulphur and iron with saline constituents in various proportions give a large choice to the invalid, who is still too apt to overlook them all and resort to foreign spas.

*Gout.* By ROBSON ROOSE, M.D. Seventh Edition. Pp. xii., 229. London: H. K. LEWIS. 1894.—This exceedingly well-written and useful monograph appears to be well up to date; but we regret to find that the author has so poor an opinion of the modern drug piperazine: from a somewhat limited experience we are inclined to think much more highly of its clinical utility, and we expect that in a future edition Dr. Roose will give it greater consideration.

*Neurasthenia and its Treatment by Hypodermic Transfusions.* By RALPH BROWNE. Pp. 56. London: J. & A. CHURCHILL. 1894.—The method of Doctor Jules Chéron, as practised at the Saint Lazare Hospital in Paris, is here described. An artificial serum, containing chloride, phosphate, and sulphate of soda, is injected hypodermically in indefinite quantity (none of the details of the method are mentioned, and we are led to assume that the injection is not to be intra-venous): the result is thus epitomised: "Bring into action, but maintain in function the great reflex of general tonicity, increasing the power of that function with each transfusion, up to the point where arterial tension has arrived at the normal level and stable conditions which are consistent with health." Why does neurasthenia any longer exist?

*Essentials of Nervous Diseases and Insanity.* By JOHN C. SHAW, M.D. Second Edition. Pp. 194. Philadelphia: W. B. SAUNDERS. 1894.—This book can only be considered a mere abstract of the chief features of nervous disease. This remark is justified when we add that the subject of insanity is dismissed in thirty-seven small pages. The rest of the book is on the same scale. To take an instance: "Paralysis of the Ocular (*sic*) Motor Nerves" occupies a little over one page, and for the treatment we learn that "Electricity is sometimes applied in these cases. If there is evidence of syphilis, large and increasing doses of iodide of potass." If, as is indicated in the preface, the book is intended for advanced students, they would certainly require more information as to the kind of electricity to be used, the strength of current, and mode of application. The bald statement that electricity is sometimes applied is worse than useless. It is almost impossible to deal at all satisfactorily with such a large subject in the way that is attempted in this book.

*The Clinical Use of Prisms and the Decentering of Lenses.* By ERNEST E. MADDOX, M.D. Second Edition. Pp. xi., 170. Bristol: JOHN WRIGHT & Co. 1893.—The first edition of this book met a recognised want; the second has been enlarged and improved. The geometrical and optical properties of prisms are simply but sufficiently discussed, their clinical uses are explained and exemplified. The way in which prisms interfere with the usual association between accommodation and convergence is described, and also their effects in the causation or relief of diplopia. The chapter on "The Study of Convergence" is interesting and complete: the author subdivides convergence into "initial" or "tonic," "accommodative," and "reflex." The anatomical position of most eyes, when freed of all nerve control, is in divergence, as shown in sleep and under anæsthesia. "Initial" convergence gives binocular vision for distance. "Reflex" aids in controlling the proper position of the eyes for all distances, through some cerebral centre which constantly is exercised to maintain vision binocular and definite. "Accommodative" effort for each dioptre should exercise about three-quarters of a metre angle of associated convergence. The practical uses of prisms both in diagnosis and treatment are discussed and summarised with much ability. Among the numerous neurasthenic patients who seek the aid of the ophthalmic surgeon a few will never find relief by the most accurate correction of their refractive errors unless in addition, anomalies of convergence and faulty muscular balance are sought for, and remedied. This volume is a trustworthy guide.

*Ophthalmic Nursing.* By SYDNEY STEPHENSON, M.B. Pp. xiii., 188. London: THE SCIENTIFIC PRESS, LIMITED. 1894.—We congratulate the author on the complete manual he has produced, one well adapted for the practical use of the educated nurse and well worthy of the careful study of the ophthalmic dresser. A knowledge of the germ theory of disease (even though it be but a theory) is a great incentive to the proper study of contagion and infection, and to the most approved methods of their prevention and treatment. Mention is made of the usual remedies for diseases of the eyes, and their modes of application are discussed. The book is full of practical hints, and "what to avoid" may be illustrated by prevention in the carrying of contagion from one patient to another, or from the patient to the eyes of the nurse herself. The preparations necessary for an operation, and the usual surgical procedures, are briefly sketched, the instruments are illustrated, and various methods of dressing and bandaging are described. A brief account of the commoner diseases of the eye is given and hints as to their nursing, examination of children, and disinfection, receive ample attention. The book concludes with a glossary of ophthalmic terms.

*Notes on Nursing in Eye Diseases.* By C. S. JEAFFRESON, M.D. Pp. vii., 90. Bristol: JOHN WRIGHT & Co. 1894.—Our author regards the opinion as prevalent, that eye cases require no special nursing, but that anyone can attend to them. The contents of his book, however, go to prove the importance of special training and knowledge for the nurse who is concerned with ophthalmic practice. There are many ways in which a good ophthalmic nurse at once shows her superiority in the eye wards over one who is merely well versed in general nursing. The book before us gives in simple but definite language the author's opinions as to what his nurse ought to know, in order properly to fulfil the responsibilities of her position. The study of the book may well be recommended to all nurses in this department of surgery; it has good illustrations and contains many useful hints; various methods of applying medicaments, bandages, and electricity are discussed. We think, even if there is no special value in antiseptics, that more rigid rules might have been given for cleanliness of instruments and of surgical dressings.

*On Blinding of the Retina by direct Sunlight: A Study in Prognosis.* By GEORGE MACKAY, M.D. Pp. 52. London: J. & A. CHURCHILL. 1894.—This excellent monograph is based upon a large number of accidents to the eyesight incurred during the observation of solar eclipses. Part I. contains an historical survey of nearly all the published cases of scotoma from the cause mentioned, and many of them are pretty fully quoted. Part II. gives full notes of seven patients who were under the author's care after the eclipses of June 17th, 1890, and of June 6th, 1891. The ophthalmoscopic appearances, the careful methods of testing for defective function, and the progress, are fully described. In Part III. the nature of the injury is clinically considered. The affected area is so minute that an accurate estimate is not obtained by test types. The scotoma is best measured at a metre distance by small white and coloured discs, and metamorphopsia by fine lines ruled 2 mm. apart—metamorphopsia seems to remain permanently in most of the cases. The sun can be looked at with impunity only by use of very dark glasses, and with a large percentage of the light refracted out of view.

*Essentials of Diseases of Eye, Nose, and Throat.* By EDWARD JACKSON, M.D., and E. B. GLEASON, S.B., M.D. Second Edition. Pp. 290. Philadelphia: W. B. SAUNDERS. 1894.—The fact that this small volume has run through its first edition shows that it has been appreciated. It is well printed, and the illustrations are numerous and well chosen. The text is arranged in the form of question and answer, and though this arrangement may be very useful to those about to enter for examinations, it is not to be commended for general reading. We dislike the growing tendency to multiply almost indefinitely the books that students are expected to read, and a good practical manual for diseases of the eye, and one for the throat and nose, should render the "essentials" superfluous.

*Clinical Diagrams, with Directions for Recording cases of Heart Disease.* By GEORGE HERSCHELL, M.D. London: BAILLIERE, TINDALL & COX. 1894.—Most physicians use some mode similar to the diagrams before us for recording the features of the cases of heart disease that come before them. These diagrams supply all that is required for records of the kind, and will doubtless be found very convenient for this purpose. They are issued with and without directions for use: the latter will be useful to those who have not been in the habit of making such records and desire to do more accurate work in the future.

*Inebriety or Narcomania: its Etiology, Pathology, Treatment, and Jurisprudence.* By NORMAN KERR, M.D. Third Edition. Pp. xxxix., 780. London: H. K. LEWIS. 1894.—This work from the pen of Dr. Norman Kerr, known so well by his connection with the Dalrymple Inebriate Home, and the part he has always taken in the proper treatment of the inebriate, has now been put before the profession in a third edition, which has been enlarged very considerably. The additions chiefly consist in (1) A Study of the Conditions of Etheromania, to which attention has been a great deal directed of late by the cases in Ireland. (2) The Medical Administration of Alcohol. (3) The question of Life Insurance in connection with Inebriates, a most important question. Another subject somewhat allied to it is also treated: "The Relation of Inebriates to the Civil Law and their Criminal Responsibilities." There is also some very interesting information given about legislation for inebriates in our Colonies, the United States, and on the Continent. The book has thus been brought into touch with our present scientific knowledge, while at the same time discussing some of the burning questions of the day.

*Caracteristica Terapéutica y Especialización Clínica de las Aguas Termales de La Garriga.* Por el Dr. M. MANZANEQUE. Barcelona: TIPOGRAFIA HISPANO-AMERICANA. 1894.—This is an address to the medical society of Cataluña on the therapeutic virtues of the baths of La Garriga, which are situated in the Province of Barcelona. The author lives at La Garriga, and receives patients who are sent to him by the medical men of the neighbourhood. These baths are hot, being of the temperature of 40° centigrade, and contain sulphur, the chlorides and carbonates of soda, potash, and iron, together with a trace of arsenic. The waters are not suitable for drinking, but are used as baths and in the form of douche, and are found to be useful in rheumatism. They also, by the sedative and emollient action on the integument which they possess, alleviate, if not cure, certain skin affections, and they appear generally to be somewhat analogous to those of Buxton and Harrogate.

*Clinical Sketches.* Edited by NOBLE SMITH. London: SMITH, ELDER & Co.—This monthly journal, beginning with the present year, makes a new departure in medical journalism. Its purpose is to supply an illustrated record, and it is intended that he who rides may read. The size is convenient, the printing is excellent, and the illustrations under the control of so excellent an artist as the editor should surpass anything recently published. The portrait of William Harvey is a good beginning to the series, which will, we trust, be long continued. The undertaking should receive the hearty support of the profession, and become a brilliant success.

*Edinburgh Hospital Reports.* Volume Second. Edinburgh: YOUNG J. PENTLAND. 1894.—When we say that the second volume of these Reports is fully equal in excellence to the first, those of our readers who studied the first volume will know that this is very high praise. These volumes must add, if that be possible, to the high reputation of the great Medical University of the North. It is impossible here to attempt to give anything like a systematic criticism of the various papers contained in this volume. We will content ourselves with indicating that there are, amongst others, interesting and important contributions to the examination of the abdomen and intestines, to the treatment of enteric fever, to the symptomatology and pathology of gastric ulcer, dermatitis herpetiformis, on a case of enlargement of liver and spleen with diminution of leucocytes, to the morbid anatomy of Graves's disease, to congenital heart disease, to tricuspid stenosis, and to incompetence of pulmonary valves. There are also papers on albuminuria, acute nephritis, rupture of the kidney, renal calculus, and several articles on rare and interesting forms of nervous disease. Surgery is represented by excellent papers on such topics as the electrolysis of vascular tumours, hints and suggestions in the practice of surgery, and notes on operative surgery. The type, paper, and general get-up of the book leave nothing to be desired, and there are numerous illustrations, many of them in colours, which are admirably executed.

*Compulsory Notification in England and Wales in* 1892. *State Aid in Relation to Port Cholera Expenses.* By ERNEST HART, D.C.L. Pp. 60. London: SMITH, ELDER & Co. 1894.—These are reprints of papers on subjects which created a good deal of interest at the time, and it will be convenient to sanitary authorities and their officers to possess them in permanent form.

*Compulsory Vaccination and Small-pox in Relation to Vaccination.* By ERNEST HART, D.C.L. Pp. 48. London: SMITH, ELDER & Co. 1894.—This is one of the most concise and helpful of the smaller works dealing with the influence of vaccination upon smallpox. Some accurate knowledge on this important point is essential for all practitioners, who are frequently called upon to meet hesitation or objection to vaccination on the part of their patients. A careful study of the few but pregnant facts adduced in this pamphlet should make it easy to persuade parents to accept the protection afforded by vaccination.

*Prescribing and Treatment for Infants and Children.* By PHILIP E. MUSKETT. Third Edition. Pp. xvii., 334. Edinburgh: YOUNG J. PENTLAND. 1894.—This is an unpretending but practical and useful little book. The information contained in it is generally trustworthy, and the advice as to treatment is judicious and well selected. Experience has enabled the author to make many additions and modifications in arrangement which increase its value and usefulness for ready reference. That a third edition has been called for in three years is the best testimony to the worth of the book. There is a good index.

*Alpine Climates for Consumption.* By HERBERT JUNIUS HARDWICKE, M.D. Pp. 65. London: J. & A. CHURCHILL. 1894.—Dr. Hardwicke has written several booklets on kindred topics. This one gives with clearness and brevity a very interesting account of the high resorts of the Engadine and the Rhone Valley. It reads like a novel, but is founded on fact and observation.

*Sperminum-Poehl in chemischer, physiologischer, und therapeutischer Beziehung.* Von Dr. G. BUBIS. Pp. 84. St.-Petersburg: BUCHDRUCKEREI VON WIENECKE. 1894.—This is an interesting though perhaps *ex parte* review of the work that has been done on the subject of spermin. The sensational announcements of Brown-Séquard have led to an examination of the chemistry of the subject, and of the base spermin, which appears to be a common product of many glands, such as the thyroid, pancreas, and spleen, and probably of other parts of the body. The question arises whether it is the ferment by which these glands carry on one part of their functions, and in the absence of which we find the cachexia which is so marked after removal of the thyroid. Whether, in fact, when we administer thyroid juice we are not merely employing spermin. Dr. Bubis has collected the literature of the subject under a systematic form. He shows that spermin is distinct from ethylenimin and piperazine, and that it exerts a powerful influence on the oxidation processes of the body, both those in the blood and those in the tissues themselves. When these tend to fail, spermin by a catalytic action reinforces them. Thus, in carbonic oxide and chloroform poisoning, spermin restores the oxidising power of the blood. Moreover, it causes the destruction and elimination of leucomaines, and thus hinders auto-intoxication. Most probably it is but one of several bodies secreted by the organism for similar purposes as a protection against the failure of the ordinary organs. When the salt is injected no local reaction occurs, in which it differs from Brown-Séquard's emulsion. It has a tonic influence on the nervous system, often removing motor disturbances and the pains of ataxia and cardiac disease, increasing both muscular force and the oxidation of nitrogenous matter. Whether administered by the mouth or by injection it is harmless, and may be used to combat cachexia produced by various diseases and bacterial poisonings. Though not a specific against any one disease, it is a valuable tonic in aiding the tissues to resist the effects of many: wherever, in fact, oxidation processes are diminished. Poehl gives by the mouth twenty-five or thirty drops of a four per cent. solution, or injects a Pravaz syringeful of a two per cent. under the skin. A summary of the results in 200 cases under various observers is added, and the biological importance of the substance is discussed with clearness and brevity.

*The Nurseries of Cholera: its Diffusion and its Extinction.* By Ernest Hart, D.C.L. Pp. vii., 35. London: Smith, Elder & Co. 1894.—This graphic and instructive address, delivered before the Section of Public Medicine of the British Medical Association at Newcastle, August, 1893, deals with the diffusion of cholera from the East, and in particular, with its diffusion through the Meccan Pilgrimage; and lays down concisely, upon a sound basis of common sense, what should be done to prevent this recurring calamity. It is well worth the thoughtful perusal of every one interested in International Preventive Medicine; and it is to be hoped that the time is not far distant at which the adoption of such measures as are indicated may become practicable. The book has in addition an Appendix of the International Cholera Convention of Paris, 1894, and on the Meccan Pilgrimage.

*Essentials of the Diseases of the Ear.* By E. B. Gleason, S.B., M.D. Pp. 147. Philadelphia: W. B. Saunders. 1894. —The only fault we can find with this small and unpretending work is that it is written in the irritating form of question and answer. The matter contained in the answers is very good and accurate. A great deal of information is compressed into a small space, and the author has drawn freely upon the best modern authorities for his facts, so as to give a trustworthy and instructive outline of the subject of diseases of the ear. The book can be recommended to students, both ante- and post-graduate.

*Myxœdema, Cretinism, and the Goitres.* By Edward T. Blake, M.D. Pp. 89. Bristol: John Wright & Co. 1894.—This book seeks to establish the following points:—1. That Graves's disease in woman is an autotoxis, most frequently caused by the absorption of purulent products; that process being aided, rather than induced, by the toxines of terror or shock. 2. The same products lead to the production of rheumatism in males, by acting in the same locality; viz., the medulla oblongata. 3. If these products abolish the functions of the thyroid, we may get myxœdema. 4. If these products invade the cortex in large quantity and abruptly, we get a psychosis, as, for example, mania; or some neurosis, such as epilepsy. 5. If the invasion be more gradual and the poisons be more diluted, we get chorea. We think all these points are not only hypothetical, but likely to remain so. The book is well got up, is easy to read, and is entertaining.

*Dissections Illustrated. Part III.—The Head, Neck, and Thorax.* By C. GORDON BRODIE. Pp. 114. London: WHITTAKER & Co. [1894].—This admirable work, which is to be completed in four parts, has been brought one step farther by the issue of the present number, which deals with the anatomy of the head, neck, and thorax. The coloured plates are twenty in number, and they are models of accuracy and clearness, while additional aids are afforded to the student by numerous diagrams interspersed between the plates. We congratulate the author and the artist (Mr. Percy Highley) on the success of their work.

*First Aid to the Injured and Management of the Sick.* By E. J. LAWLESS, M.D. Pp. xvi., 262. Edinburgh: YOUNG J. PENTLAND. 1894.—This book contains a good deal of old matter put in a clear way; it will be found useful to those giving ambulance instruction, as well as to pupils for home study. We think the appendix to Part I. might have been omitted, as we consider printed questions and answers lead to a system of cramming. The book is well printed, the illustrations are good of their kind, and the full index will be found valuable.

*The Pharmacopœia of the Hospital for Diseases of the Throat.* Fifth Edition. Pp. 112. London: J. & A. CHURCHILL. 1894.—The introduction of various new remedies and modifications in the treatment adopted at this hospital rendered it necessary to make considerable alterations in the formulæ of earlier editions, for which the late Sir Morell Mackenzie was mainly responsible. The book will be found very useful as a guide to the most appropriate remedies in the various affections of the throat and nose.

*Transactions of the Ophthalmological Society of the United Kingdom.* Vol. XIV. London: J. & A. CHURCHILL. 1894.—This volume contains many communications of general medical interest. Notable among these are the papers of Dr. James Taylor on "Optic Neuritis in its Relation to Intra-cranial Tumour and Trephining," and Mr. Jonathan Hutchinson's on "School Ophthalmia." The debates which followed these papers are also reported. Many cases are recorded of most of the exceptional diseases of the eye to which recent attention has been paid.

*A Clinical Manual.* By ANDREW MACFARLANE, M.D. Pp. xii., 139. New York: G. P. PUTNAM'S SONS. 1894.—In accordance with the fashionable practice of boiling down to concentrated extracts, the author has condensed the work of the physiologist and the bacteriologist, and has given a concise account of the methods which have shewn themselves to be practicable and useful to the clinical physician. These concentrated products are not always easy of digestion: we prefer them diluted: nevertheless the book appears to us to be an excellent epitome of the larger works on clinical diagnosis.

*Asthma and Chronic Bronchitis.* By JOHN C. THOROWGOOD, M.D. Pp. 136. London: BAILLIERE, TINDALL & COX. 1894.—This little volume is a new edition of the author's *Notes on Asthma and Bronchial Asthma.* He gives an excellent outline of what is known on the subject, and illustrates the effects of treatment by numerous cases. Case XII. derived benefit from iodide of potassium in five grain doses: this is the only mention of this drug throughout the book. We searched for information on grindelia robusta and the euphorbia pilulifera: both are cursorily mentioned, but the author appears to have had little experience of either. He relies more on arsenic than any other drug, and has not even mentioned "Himrod" and other powders which are in daily use.

*Elements of Surgical Pathology.* By AUGUSTUS J. PEPPER, M.S., M.B. Fourth Edition. Pp. xvi., 607. London: CASSELL AND COMPANY, LIMITED. 1894.—The fact that this little volume has reached its fourth edition attests its popularity. There is an increase of 75 pages and 15 woodcuts over the last edition, and some new subjects—*e.g.*, acromegaly, mycetoma, myositis ossificans—are considered. There is little to find fault with in the work, except some disproportion in the descriptions of various diseases: thus acromegaly is favoured with three pages, whilst so important and common an affection as appendicitis gets barely twelve lines. We should also have liked to have seen the subject of hernia treated at somewhat greater length. Again, it would perhaps have been better to have devoted a separate chapter to diseases of the breast. The word "breast" is not to be found in the index, and its diseases are described under various titles throughout the volume.

*The Album.* No. 1, February 4th, 1895. London: THE "ILLUSTRATED LONDON NEWS" OFFICE.—There seems to be no limit to the ingenuity of device which the purveyors of periodical, and especially illustrated, literature display. In this excellently carried out design the idea is to "take a topic of the day, or a distinguished personage . . . and give him, or the topic, sixteen pages of pictures," and in addition to provide a supplement of like size dealing with a special study of Art or Nature. All this is rendered possible by the development of photography and process-work. The illustrations in the number which has been sent us are admirable.

*The International Directory of Secondhand Booksellers.* Rochdale: JAMES CLEGG. 1894.—This work provides a mass of information interesting to the bibliophile, although we are sorry to see that this issue does not contain some useful things of which we gladly availed ourselves on former occasions. There are still some noteworthy omissions in the Bibliographical Works of Reference. We should like to see the publication made an annual one.

*Pocket Therapeutic Notes*. Second Edition. Bristol: FERRIS AND COMPANY. 1894.—This neat and well-printed pocket companion has been well revised, and gives much useful information on the new drugs which are worth knowing. The list of coated pills is very complete; but we often find that the coating materially interferes with the efficacy of the contents. The soluble gelatine capsules are becoming more and more popular, and the list is now largely increased. These and the long list of compressed tabloids must have greatly simplified the process of dispensing. An alphabetical index of diseases and treatment, in eighteen pages, may satisfy inquirers who have no time to consult more trustworthy information.

